# Initial Formulation of Novel Peanut Butter-like Products from Glandless Cottonseed

**DOI:** 10.3390/foods12020378

**Published:** 2023-01-13

**Authors:** Zhongqi He, Huai N. Cheng, Jibao He

**Affiliations:** 1 USDA-ARS, Southern Regional Research Center, New Orleans, LA 70124, USA; 2 Coordinated Instrument Facility, Tulane University, New Orleans, LA 70118, USA

**Keywords:** butter, cottonseed, glandless, scanning electronic microcopy, spreadability, texture

## Abstract

Glandless (Gl) cottonseed is a unique cotton variety with only a trace content of toxic gossypol present. This new cottonseed raises the potential of its enhanced utilization as an agro-food for human consumption. In this work, Gl cottonseed kernels were used with additional cottonseed oil to produce novel peanut butter-like products. Kernels roasted at two temperatures (140 or 150 °C) for a given time (15 or 30 min) were first ground with different ratios of cottonseed oil and two other ingredients (i.e., salt and sugar) with a food blender, and then passed through a meat grinder with a 4-mm-hole grinding plate. Per the preliminary result, the butter-like products with Gl kernels roasted at 150 °C were subject to further structural and textural evaluation. The color of the two butter-like products was comparable to a commercial peanut butter, but the formers’ textural properties were significantly different (*p* ≤ 0.05) from the latter. Morphologic examination by Scanning Electron Microscopy (SEM) and cryo-SEM revealed that the butter product with a longer (30 min) roasting time possessed a smoother surface than the products with a shorter (15 min) roasting time. Oil stability test showed no substantial oil separation (<3%) from the butter products over 7 weeks at ambient temperature (22 °C). This work provides the basic information and parameters for lab cottonseed butter making so that optimization and characterization of cottonseed butter formation can be designed and performed in future research.

## 1. Introduction

As the word “butter” was derived from Greek bou-tyron (cow cheese), animal butters, especially dairy butter, are the traditional butter products. However, plant-based (nut and seed) butters have steadily increased in consumer popularity. Currently, there are at least eight types of plant-based butters or spreads reported in literature [1,2,3]. These plant nuts and seeds are almond, cashew, hazelnut, sunflower, sesame, peanut, pistachio, pumpkin seed and soy. As it is the most commercially available, peanut butter is frequently used as a reference for comparison in the evaluation of other plant-based butters [4,5]. While many of the plant-based butters are prepared with whole kernels, pumpkin seed based products are made from hull-less pumpkin seed press-cake or flour, which are the by-product of the pumpkin seed oil process [6,7]. Plant-based butters are generally prepared by roasting and grinding with some additives [8,9]. The temperatures for roasting and grinding are both vital for the formulation of healthy and nutritious plant-based butters [10]. Oil is an essential component of butter products to create butter products with the proper consistency and stability. As pumpkin seed-based products are made from hull-less pumpkin seed press-cake or flour, hemp oil in the range of 20–40% was added to produce desirable pumpkin seed-based spreads [6]. In addition to adjustments of the oil types and contents, sweetener, salt, stabilizer, and/or enhanced protein sources are also included in the butter formulations [3,9]. To provide important information to the product developers, plant-based butter products are routinely evaluated by microstructural and textural methods [3,9,11,12]. For example, one of the textural parameters (spreadability) of hazelnut spreads was found to be negatively related to the consistency and flow indices determined by rheology, and the meltability was found to be strongly dependent on measured thermal parameters [12].

Due to of the presence of gossypol higher than the US FDA’s food limit of 450 ppm, glanded cottonseed are typically not suitable for human consumption [13]. The discovery of a glandless (Gl) mutant in the 1950’s made the food utilization of cottonseed possible [14,15]. However, there are few publications on the formulation of cottonseed products such as edible nuts, brownies, cookies and as a component in corn tortillas, and these products have not been fully evaluated [16,17]. Recently, the use of modern genetic modification technologies has enabled several new Gl and low-gossypol cotton lines to be created [13,18]. The exploration of these Gl cottonseeds as food products and functional food supplements would greatly enhance the economic impacts of these new cotton lines [19,20,21,22]. We hypothesized that (1) the Gl cottonseed kernels could be used to formulate peanut butter-like spread products for human consumption, and (2) external oil would be needed for preparation, as cottonseed kernels contain less oil than peanuts. Thus, this work attempted to gather the basic information and manufacturing parameters for novel peanut butter-like products from Gl cottonseed so that relevant experiments on optimization and characterization of cottonseed butter can be designed and performed later. These butter-like products were characterized for selected textural, physical, and color properties through both in-house instrumentation and outside collaboration.

## 2. Materials and Methods

### 2.1. Materials

Gl cottonseed from the NuMex series was provided by Cotton, Inc. (Cary, NC, USA). These seeds were dehulled mechanically by cracking with a 20.32-cm plate mill and then separated with a vibratory shaker. The kernel products were further cleaned by passing the material through a laboratory aspirator to remove the non-kernel material. The selected chemical composition of the Gl kernels is listed in Table 1.

Cottonseed oil (Admiration Foods, Englewood, NJ, USA) and commercial Skippy creamy peanut butter (Hormel Foods Corporation, Austin, MN, USA) were acquired from local stores.

### 2.2. Butter-like Product Preparation

The Gl kernels were used as the base material (70.0, 75.0, and 80.0%) to produce peanut butter-like spread formulations. Cottonseed oil (11.8, 16.8%, and 21.8%), cane sugar (7.5%), and table salt (0.7%) were the other three ingredients used in the formulation. The Gl kernels were first roasted in a convection oven (Thermo Scientific Precision Compact Ovens, Waltham, MA, USA) at 140 or 150 °C for a given time (15 or 30 min) [24]. The roasted kernels were then ground with Waring Commercial Blender (Model WF2211214, Torrington, CT, USA) at a high speed for 3 min, then mixed thoroughly with the three additives with a spatula. The mixture was then passed through a Smokehouse meat grinder with a 4-mm hole plate (Buchanan Dam, TX, USA). The extruded products were then visually examined. There were a total of eight preliminary trials with selective combinations of formulations and roasting conditions. Per these trials, two products (B15 and B30) roasted for 15 and 30 min at 150 °C were created for the initial characterization. Those samples were kept at 4 °C until analysis.

### 2.3. Color and Water Activity Measurements

The color of the raw materials and products was analyzed with a Spectro2guide spectrophotometer with built-in calibration, standard in the docking station (BYK-Gardner, Columbia, MD, USA) and recorded on the CIE L*a*b* color coordinates [7]. Parameters determined were the L* value (lightness/darkness), a* value (greenness/redness), and b* value (blueness/yellowness) [3]. The analysis was replicated 3 times.

Water activity (a_w_) was measured at 25 °C on triplicates of ground kernels and butte samples with laboratory water activity meter (Lab Touch-aw, Neutech group, Farmingdale, NY, USA).before the measurement, the meter was calibrated with SAL-T58 salt standard provided by the meter manufacturer.

### 2.4. Microstructural Imaging Analysis

For scanning electron microscopy (SEM), oil in the butter products was first removed by hexane extraction to reduce its possible interferences in the SEM imaging process [10]. During analysis, a thin layer of the de-oiled butter sample was gently attached to an 8 mm × 12 mm double-sided sticky carbon tape on an aluminum stud. The butter sample was coated with 3-nm thickness of carbon using a Cressington 208HR sputter coater. The samples were observed and imaged with a Hitachi S-4800 Field Emission Scanning Electron Microscope (Hitachi, Japan), operating at 3 kV [23,25].

Cryo-SEM was applied for non-deoiled butter samples. The Gatan Alto 2500 cryogenic system and the Hitachi S-4800 field emission SEM were used for acquiring cryogenic SEM images. A 5-uL cream sample was loaded to a sample holding device, a 3-mm diameter cylinder, and formed a crown-like cap on the top of the cylinder. The sample was plunged into a cup of slushed liquid nitrogen and vitrified. A fracture surface of the sample was acquired at −130 °C, sublimed 5 min at −95 °C, and coated with Pd/Pt alloy at −130 °C. The fracture surface was observed at 3 kV at −130 °C.

### 2.5. Texture Analysis

The butter products were evaluated at 22 °C for firmness, spreadability and adhesiveness with an EZ-SX texture analyzer (Shimadzu Scientific Instruments, Columbia, MD, USA). A spreading jig set was used to measure the test force required to spread a butter sample between the upper and lower jigs. The cone was lowered at 5 mm s^−1^ to a penetration distance of 2 mm, the raised at 5 mm s^−1^ [5].

### 2.6. Butter Stability

The butter (oil) stability was determined with accelerated oil separation rates [8,9]. The tested butter products (5.000 g each) were put into capped 50-mL centrifuge tubes and kept at room temperature (22 °C). After storage for a given time, the butter tubes were centrifuged at 1258× *g* for 10 min at 22 °C. The surface oil was removed after centrifugation and the percent separation was calculated below:

Oil separation content (%) = Oil separated after centrifugation (g)/5.000 (g) × 100

## 3. Results and Discussion

Preliminary butter making trials were conducted at three levels of Gl kernels (70.0, 75.0, and 80.0%), three levels of oil (11.8, 16.8%, and 21.8%), and fixed levels of sugar (7.5%) and salt (0.7%) with roasting conditions at 140 or 150 °C. Visual examination of the products indicated that the mixture of 75.0% cottonseed kernels and 16.8% oil possessed a more appealing, peanut-like appearance and textural characteristics at either temperature. Thus, this formulation was adopted to produce two cottonseed butter-like samples (i.e., B15 and B30 with Gl kernels roasted at 150 °C for 15 and 30 min) for its physical and textural evaluation.

### 3.1. Color and Water Activity

**Figure 1:**
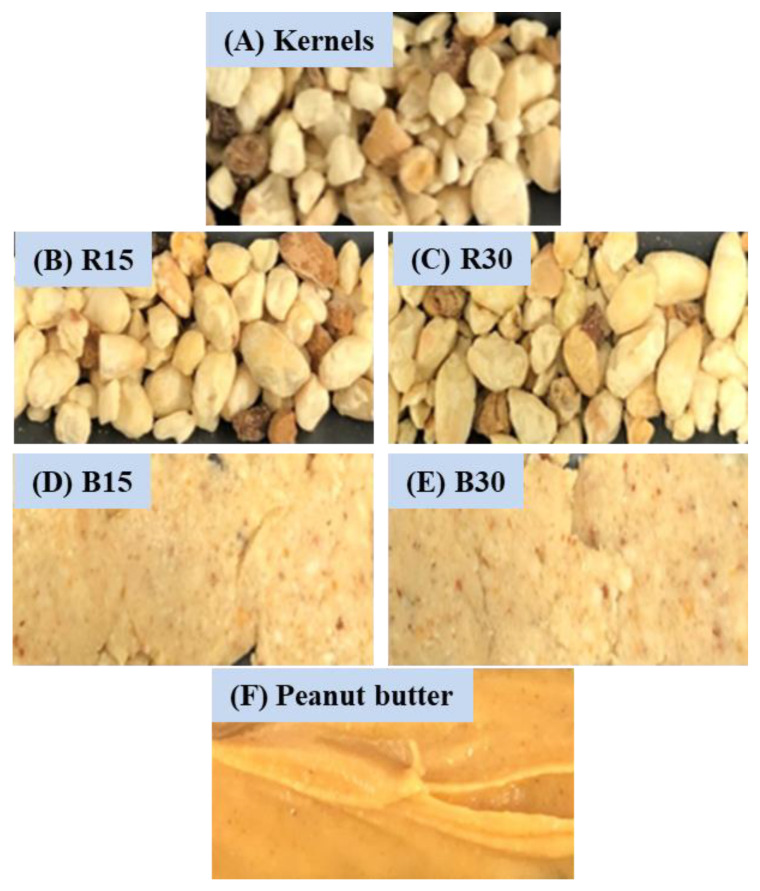
Images of unroasted glandless cottonseed kernels (**A**), kernels roasted at 150 °C for 15 min (R15) (**B**), 150 °C for 30 min (R30) (**C**), their corresponding butter products B15 (**D**) and B30 (**E**), and commercial Skippy creamy peanut butter (**F**).

The images of the unroasted and roasted cottonseed kernels are shown in Figure 1. Visually, unroasted kernels were light brown. The color of the butter products was similar to that of the roasted kernels but appeared more homogenous. Compared with 15-min roasting, a longer roasting time (i.e., 30 min) did not seem to make much of a difference in the color the whole kernels nor the butter products. Visually, the colors of both cottonseed butter products were lighter than the commercial Skippy creamy peanut butter.

The quantitative colorimetric profile of these samples is presented in Table 2. The specific hue parameters (a* and b*) both steadily increased following the roasting process and butter formulation. The a* parameter shows the balance between red (positive values) and green (negative values), and the b* parameter is for yellowness. Thus, while the color of all samples was yellow and reddish, the tone moved after roasting and butter blending. The peanut butter, in comparison, had high values of both a* and b* parameters, rendering the sample more reddish. On the other hand, the comprehensive lightness (L* values) did not change much over the processing procedure. The L* values of the two butter products were 57.18 and 55.55, respectively; comparable to that of peanut butter. These values were similar to those of peanut butter and pumpkin seed spread [7,26]. Indeed, Pattee et al. [26] suggested that L* values of 58–59 are optimal for roasting for peanut butter production, although lower L* values are reported in some peanut and other plant-based butter products [5,9].

The water activity of unroasted kernels was 0.570. The activity values decreased after roasting and butter formulation significantly at *p* < 0.05 (Table 2). It was reported that a significant (*p* < 0.05) a_w_ decreased after 30 min of roasting hazelnut samples [27]. Similarly, a longer roasting time (30 min) seemed to further lower the water activity in the Gl cottonseed kernel and butter samples, even though the water activities (0.543 and 0.530) of the two cottonseed butter samples were much higher than that (0.253) of the commercial peanut butter in comparison. While lower water activity is favorable to prevent microbial growth during storage, biochemical and microbiological reactions in a food system can be inhibited and the deterioration of the product prevented when water activity is < 0.6 [28]. It was also reported that the optimum water activity for the storage of macadamia nuts is < less than 0.53 at 25 °C [29,30]. Thus, the water activity values of these cottonseed butter products were in the acceptable range. Further lowering the water activity values may be reached with the addition of additives and stabilizers as in commercial peanut butter [31]. A moisture sorption isotherm study of these cottonseed products will provide more insight on the long-term storage of these cottonseed products [28,30,32].

### 3.2. Microstructural Analysis

**Figure 2:**
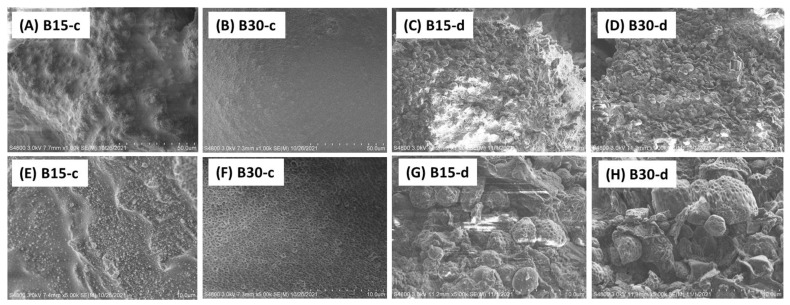
SEM images of cottonseed butter products with 15 (B15) and 30 min (B30) kernel roasting at 150 °C. Suffixes “c” and “d” indicate “cryo-SEM” and “de-oiled” samples, respectively. Bar distance is 50 and 10 µm, respectively, for upper and lower rows.

The two butter products of optimal formulation with cottonseed kernels roasted at 150 °C for 15 and 30 min were examined via scanning electronic microcopy (Figure 2). The microstructural features of the Cryo-SEM images consisted of broken cell wall fragments, protein bodies, and starch granules as observed in peanut butter samples [8,33]. Differences between the Cryo-SEM images of the two products were observed. The morphology of the butter product with a 15 min roasting time (i.e., B15-c in Figure 2A,E) was more similar to a rough sponge structure filled with many tiny particles. The sponge structure could be assigned the carbohydrate sheet with continuous oil phase, and the tiny particles were clumps of protein bodies [33]. In contrast, the surface of the butter sample with 30 min roasting (B30-c, Figure 2B,F) was smoother, and the tiny particles were distributed more evenly on the surface.

After de-oiling, the images of the butter samples looked rougher and contained irregular spherical, ellipsoidal, and flat particles (B15-d and B30-d). The images in the upper row, with a 50-μm bar distance (Figure 2C,D) looked similar to those of fish meal prepared with glandless cottonseed meal [34]. The spherical and ellipsoidal particles could be assigned to starch granules, and the large flat and round particles could be proteins of the cottonseed. Further examine of the two butter samples at a higher magnification (Figure 2G,H) revealed the rough and /or pit-filled features of these particles, similar to previous observations of defatted cottonseed and soy meal products [35,36]. This differential features in surface smoothness before and after de-oiling showed the critical role of the oil component in smoothing and stabilizing oil seed products [23,33]. In addition, the images of the two de-oiled butter samples (B15-d and B30-d) looked more similar to each other. Those observations indicated that the roasting time seems to have more impact on the oil-related morphology of the cottonseed butter products. In addition, the SEM images also revealed that the morphology of the two butter products of cottonseed was not as smooth as that of peanut butter by comparison with the relevant SEM images in the literature [8,10].

### 3.3. Texture Characteristics

**Figure 3:**
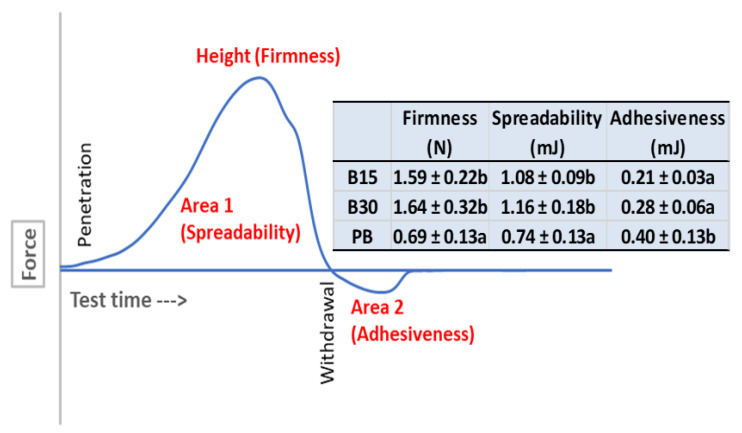
The characteristic force–deformation curve of butter samples and the measurement of the three texture parameters. B15 and B30, cottonseed butter made from glandless cottonseed kernels roasted at 150 °C for 15 and 30 min, respectively. PB, commercial Skippy creamy peanut butter. Data are present in the format of average ± standard deviation (*n* = 3). Different letters in the same column indicate these values statistically significantly different (*p* ≤ 0.05).

The force–deformation curve of the texture analysis is shown in Figure 3. Three texture parameters were derived from the curve. They are (1) firmness measured as the peak force reached during compression (height H), (2) spreadability as the positive work done (Area 1), and (3) adhesiveness of the work to pull the matching probes apart (Area 2) [5,8]. The values of all three parameters of the B30 butter samples were higher than those of the R15 samples. However, the differences were not statistically significant (*p* > 0.05). In other words, the impact of the roasting time increase from 15 min to 30 min was minimal. In comparison, the three texture parameters of the commercial peanut butter were significantly (*p* ≤ 0.05) different from those of cottonseed butters. The values of firmness and spreadability of peanut butter were higher than the two cottonseed butter products; however, the adhesiveness of the former was lower than the latter. These differences indicated that the cottonseed butter products were stiffer and less sticky than the peanut butter, in comparison. Further refinement of the cottonseed butter formulation with higher oil content and/or additive inclusion may improve the softness and spreadability as those they are for peanut butter and other plant butter products [5,6,8,9].

### 3.4. Butter Stability

**Figure 4:**
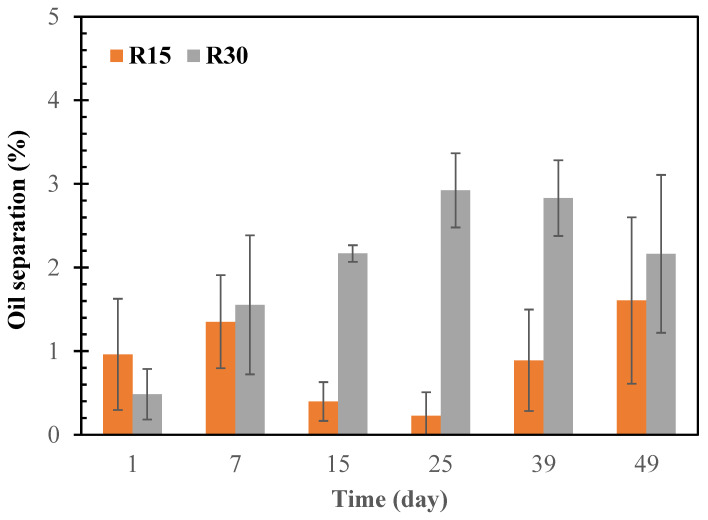
Changes in the rate of oil separation from the cottonseed butters during 49 days of storage at room temperature (25 °C). B15 and B30, cottonseed butter made from glandless cottonseed kernels roasted at 150 °C for 15 and 30 min, respectively. Data are presented as averages with standard deviation bars (*n* = 3).

The stability of the cottonseed butter products was evaluated by oil separation during the storage of the butter products at room temperature over 49 days (Figure 4). During the storage time, the oil separated from the butter bodies was not more than 3% of the butter weight, while the total oil content (cottonseed oil fraction and added oil) was 43.1% of the butter weight. This rate of oil separation was much lower than those in the literature for other types of butter products, such as 11–13% separation in peanut butter [8], 15–30% in walnut butter [9], and 11% in sesame paste [37]. Similar to the oil loss of walnut butter [9], the oil separation of the cottonseed butter fluctuated with storage time, but with a general trend of increasing oil loss with increasing storage times. The longer roasting duration of cottonseed kernels resulted in more oil loss from the butter during storage. The maximal oil loss during the storage was 1.6% and 2.9% of the total butter weight, respectively, for B15 and B30 samples. Thus, stabilizer additives will not be required for formulation of the cottonseed butter under current preparation conditions.

## 4. Conclusions

Glandless cottonseed kernels can be used to make novel peanut butter-like food products. An initial workable formulation included 75.0% roasted cottonseed kernels, 16.8% cottonseed oil, 7.5% sugar and 0.7% salt. The color of the cottonseed products was comparable to peanut butter. However, texture measurements indicated that the cottonseed butter products were stiffer with harder spreadability in comparison with a commercial peanut butter. Microstructural analysis revealed that the butter product with a longer (30 min) roasting time possessed a smoother surface than the products with a shorter (15 min) time. On the other hand, the butter product with a 30 min roasting duration showed a higher oil separation rate (up to 2.9%) than the product with 15 min roasting time (1.6%). Nonetheless, the oil separation during the storage of both cottonseed butter products was lower than those of peanut butter and walnut butter as reported in the literature, indicating a higher butter stability of the cottonseed butter products. This study demonstrates the potential of glandless cottonseed in developing novel food products, and may serve as the basis of refining cottonseed butter formulations in future research.

## Figures and Tables

**Table 1:** Selected chemical components of glandless cottonseed kernels. ADF, acid detergent fiber. ADL, acid detergent lignin. Adapted from [23].

Major Component (g·kg^−1^)						
Moisture	Gossypol	Oil	Protein	ADF	ADL	Starch
68.3	0.06	350	421	109	67.8	16.6
**Macro element (g·kg^−1^)**						
P	Ca	K	Mg	Na	S	Ash
11.5	2.3	12.8	6.1	0.6	4.9	47.9
**Trace element (mg·kg^−1^)**						
Fe	Zn	Cu	Mn	B	Ni	Al
111	74.3	19.0	13.3	14.2	2.1	109.8

**Table 2:** Colorimetric parameters (L*, a*, and b*) and water activity (a_w_) of unroasted glandless cottonseed kernels, kernels roasted at 150 °C for 15 (R15) and 30 (R30) min, and their corresponding butter products (B15 and B30). Data of commercial Skippy creamy peanut butter (PB) was measured for comparison. Data are present in the format of average ± standard deviation (*n* = 3).

	L*	a*	b*	a_w_
Kernels	55.94 ± 0.04 c ^1^	0.69 ± 0.01 a	16.10 ± 0.10 a	0.570± 0.004 d
R15	58.02 ± 2.36 cd	1.63 ± 0.47 b	20.14 ± 2.99 b	0.569 ± 0.006 d
R30	60.31 ± 0.01 d	3.68 ± 0.01 d	24.36 ± 0.01 c	0.553 ± 0.004 c
B15	57.18 ± 0.02 d	3.51 ± 0.01 c	24.52 ± 0.01 d	0.544 ± 0.005 c
B30	55.55 ± 0.02 b	3.85 ± 0.01 e	24.52 ± 0.03 d	0.530 ± 0.002 b
PB	51.82± 0.01 a	10.96 ± 0.01 f	31.4 ± 0.00 e	0.253 ± 0.036 a

^1^ Different letters in the same column indicate these values statistically significantly different (*p* ≤ 0.05).

## Data Availability

The data presented in this study are available wholly within the manuscript.
